# Botulinum neurotoxin type A in the treatment of classical Trigeminal Neuralgia (BoTN): study protocol for a randomized controlled trial

**DOI:** 10.1186/s13063-015-1052-z

**Published:** 2015-12-03

**Authors:** Jan Burmeister, Dagny Holle, Eva Bock, Claudia Ose, Hans-Christoph Diener, Mark Obermann

**Affiliations:** Department of Neurology and Headache Center, University Hospital Essen, Hufelandstraße 55, 45122 Essen, Germany; Center for Clinical Trials, Essen (ZKSE) and Institute for Medical Informatics, Biometry and Epidemiology (IMIBE), University Hospital Essen, Hufelandstraße 55, 45122 Essen, Germany

**Keywords:** Trigeminal neuralgia, Botulinum toxin type A, Prophylactic treatment, Clinical trial, Prospective study, Study protocol

## Abstract

**Background:**

Trigeminal neuralgia is characterized by paroxysmal facial pain attacks. Adequate prophylactic drug therapy is often limited by the lack of efficacy and intolerance due to central nervous system side effects.

Subcutaneous injections of botulinum toxin type A are a promising treatment option for patients with unsatisfactory response to drug therapy or neurosurgical intervention. Its effects are expected to last for at least 3 months, so it could be a potential long-term treatment.

This is the study protocol of a prospective, placebo-controlled, double blind clinical trial investigating the add-on therapy of subcutaneous administration of botulinum toxin type A injections to standard treatment in therapy-refractory classical trigeminal neuralgia.

**Methods and design:**

BoTN is a prospective, double blind, placebo-controlled trial with a randomized withdrawal design in which a single blind phase is followed by a double blind phase (see also Methods and design). Eligible patients with classical trigeminal neuralgia who are otherwise refractory to medical and neurosurgical treatment will receive subcutaneous injections of botulinum toxin type A into injection sites of the affected trigeminal branch.

In the first phase all patients will receive botulinum toxin type A in a single blinded intervention. Twelve weeks later therapy responders will be allocated to the *verum* or placebo (saline) arm in a double blind, randomized manner. These injections will be performed at the same sites as the first injections.

This trial will be conducted in a tertiary outpatient clinic specialized in the treatment of headache and facial pain. There will be three investigators performing the injections who are experienced in the treatment of headache and facial pain and trained in botulinum toxin type A injections.

**Discussion:**

BoTN is designed to assess the efficacy and safety of subcutaneous botulinum toxin type A injections in addition to standard prophylactic treatment in therapy-refractory trigeminal neuralgia.

**Trial registration number:**

EU Clinical Trials Register: EudraCT-No: 2014-001959-24 https://www.clinicaltrialsregister.eu/ctr-search/rest/download/trial/2014-001959-24/DE

Date of trial registration

26 August 2014

**Electronic supplementary material:**

The online version of this article (doi:10.1186/s13063-015-1052-z) contains supplementary material, which is available to authorized users.

## Background

Trigeminal neuralgia (TN) is a chronic disorder characterized by paroxysms of unilateral, electric shock-like pain in the distribution of one or more branches of the trigeminal nerve [1].

**Table 1:** Visit schedule and methods

		First part: single blind		Second part: double blind, randomized		
Visit schedule and methods	V-1 (Screening, 8 days before V0)	V0 = open-label *verum* injection	V1 (28 ± 3 days after V0)	V2 = randomized, doubleblind intervention (84 ± 3 days after V0)	V3 (28 ± 2 days after V2)	V4 (84 ± 3 days after V2)
Patient’s consent	X					
Inclusion/Exclusion criteria	X					
Standardized, semi-structured patient history	X	X	X	X	X	X
SF-12 Questionnaire assessing life quality	X	X	X	X	X	X
ADS Questionnaire assessing depression	X	X	X	X	X	X
HIT-6 Questionnaire assessing pain severity	X	X	X	X	X	X
Neurological examination	X		X	X	X	X
Medical examination	X		X	X	X	X
Blood tests	X					
Pain-evoked potentials		X	X	X	X	X
Randomization				X		
BT-A/Placebo injection		X (*verum*)		X (*verum*/placebo)		
Assessment of baseline TN frequency		X				
Assessment of therapy response			X		X	
Assessment of adverse events		X	X	X	X	X
Pseudonymization	Patients are assigned to a screening number			Screening numbers are assigned to randomization list		

*ADS* Allgemeine Depressionsskala: General Depression Scale, *HIT-6* Headache Impact Test-6, *SF-12* 12-item short form questionnaire, *TN* trigeminal neuralgia

It is estimated that approximately 4–28.9/100,000 people worldwide suffer from TN. It affects mainly the older population and is more common amongst women [2, 3]. The underlying cause of classic TN, as defined by the ICHD-3 beta (International Classification of Headache Disorders, third edition), is assumed to be neurovascular compression caused by cerebral vessels. In particular the superior cerebellar artery can locally affect the trigeminal nerve causing segmental demyelination and consecutively ephtatic conduction of painful paroxysms [3].

Secondary forms caused by tumor, trauma, multiple sclerosis or post-herpetic neuralgia exist and are classified by the ICHD-3 beta as secondary painful trigeminopathies.

Single pain episodes of TN only last for a few seconds. The therapeutic strategy is prophylactic treatment. Treatment consists of anticonvulsive drugs such as carbamazepine (CBZ), oxcarbarzepine (OXC), phenytoin or gabapentin. Major treatment limitations are lack of efficacy and, in an older-aged population, intolerability due to central nervous system side effects. Drug refractory courses affect many patients [4].

Evidence for the efficacy of interventional procedures such as gamma-knife surgery, microvascular decompression and percutaneous Gasserian ganglion intervention is limited and sensory side effects in the affected trigeminal branches are common [5].

Botulinum neurotoxin type A (BT-A) is a potent neurotoxin derived from *Clostridium botulinum*. It blocks the release of acetylcholine at the neuromuscular junction and has also been found to inhibit the release of proinflammatory peptides. In addition BT-A binds to C- fibers. Analgesic efficacy is proven in the treatment of chronic migraine and its antinociceptive effects on neuropathic pain have been established in animal models [6].

Subcutaneous injections of BT-A are a non-invasive treatment option, which is suitable for patients where drug therapy or neurosurgical intervention have failed. Its clinical effects on TN have been studied in several open and one double blind trial [7–12].

This trial will be conducted in a tertiary outpatient clinic specialized in the treatment of headache and facial pain.

### Standard medical treatment

First-line treatment agents in the therapy of TN are CBZ and OXC [13, 14].

CBZ has proven its efficacy in four randomized controlled trials (RCTs) and is approved for TN [15–18]. It is estimated that only 50 % of the patients remain long-term responders. Therapy-limiting side effects are common. In doses higher than 600 mg/d central nervous system side effects such as ataxia, fatigue and vertigo are very common. Auto- induction of metabolic liver enzymes can lower plasma CBZ levels and lead to numerous drug interactions.

In terms of efficacy oxcarbazepine is assumed to be comparable to CBZ but with a more favorable risk profile. Due to the lack of sufficient clinical data the evidence for its efficacy is limited and it can only be prescribed off-label. Under therapy, regular screening for hyponatremia is necessary [19, 20].

Second-line agents are the GABA-B-receptor-agonist baclofen and other anticonvulsive drugs such as phenytoin, gabapentin, lamotrigine, levetiracetam, pregabalin, topiramate, valproic acid with either insufficient clinical data or a less favorable risk profile [21–32].

All the aforementioned substances and combinations of substances can be taken for the duration of the clinical trial as long as the dosage remains unchanged.

### Neurosurgical procedures

Neurosurgical procedures are considered alternative treatment options for patients with drug refractory courses:

- Microvascular decompression (Janetta procedure) is estimated to have a response rate of 68 % after ten years and is preferred over neuroablative procedures because trigeminal function will be preserved [33]
- Percutaneous ablative procedures such as thermoablation of the Gasserian ganglion is estimated to have a 5-year response rate of 50 % and moderate to severe sensory side effects such as trigeminal hypoesthesia, or anesthesia dolorosa [34–36]
- Radiosurgical treatment with a gamma-knife has a lower response rate (41–45 % after 5 years) with a more favorable risk profile (sensory impairment in 7.7–49 %) [37]

### Previous studies of BT-A in trigeminal neuralgia

The efficacy of BT-A in classical TN has been investigated in several open-label studies and one RCT [7–12]. There were considerable variations in dosage (6–100 U BT-A per intervention) and the number/localization of injection sites (between 2 and 15).

Wu et al. conducted the sole RCT [7]: 42 patients were allocated to either intradermal and optionally, if the second trigeminal branch was affected, submucosal injection of 75 U BT-A at 15 injection points in the area where the patient experienced pain. A response rate of 68 %, defined as > 50 % visual analogue scale (VAS) score reduction (as compared to 15 % in the placebo group) has been reported after 12 weeks.

Hu et al. reviewed the previous studies and rated the current evidence grade Ib. Further controlled clinical trials were recommended. Overall response rates, defined a > 50 % reduction in pain frequency or VAS scores were higher than 60 % [38].

## Methods and design

### Study design/general structure

The study design is prospective, randomized, placebo-controlled and double blind (Table 1). A randomized withdrawal design was chosen, which means that all patients who respond positively to the first treatment intervention will consecutively be randomized to continuing the intervention or receiving placebo. This study design is useful in clinical trials with only a small number of patients in order to achieve the necessary statistical power. Since all patients will at least once receive *verum* injections a lower dropout rate can be expected as compared to a study in parallel group design.

The study consists of three phases: baseline phase, single blind phase and double blind phase. Study duration for a single patient will be, depending on therapy response, between 5 and 25 weeks.

During the baseline phase (week −1) TN attack frequency and pain intensity scores (numeric rating scale, NRS) will be assessed with a specific diary. Patients are required to keep their concomitant TN medication (see also “concomitant medication”) unchanged. Patients with an average frequency of more than three daily paroxysms will enter the open-label phase of the trial.

### Estimated timeline

Recruitment will begin in November 2014 and will end in July 2016.

### Intervention

During the single blind phase (weeks 1–12) all eligible patients will receive BT-A injections in accordance to the injection scheme given below. After week 4 non-responders to treatment will finish the study early. Frequency reduction of less than 30 % as compared to baseline phase will be considered a non-response to BT-A.

**Fig. 1:**
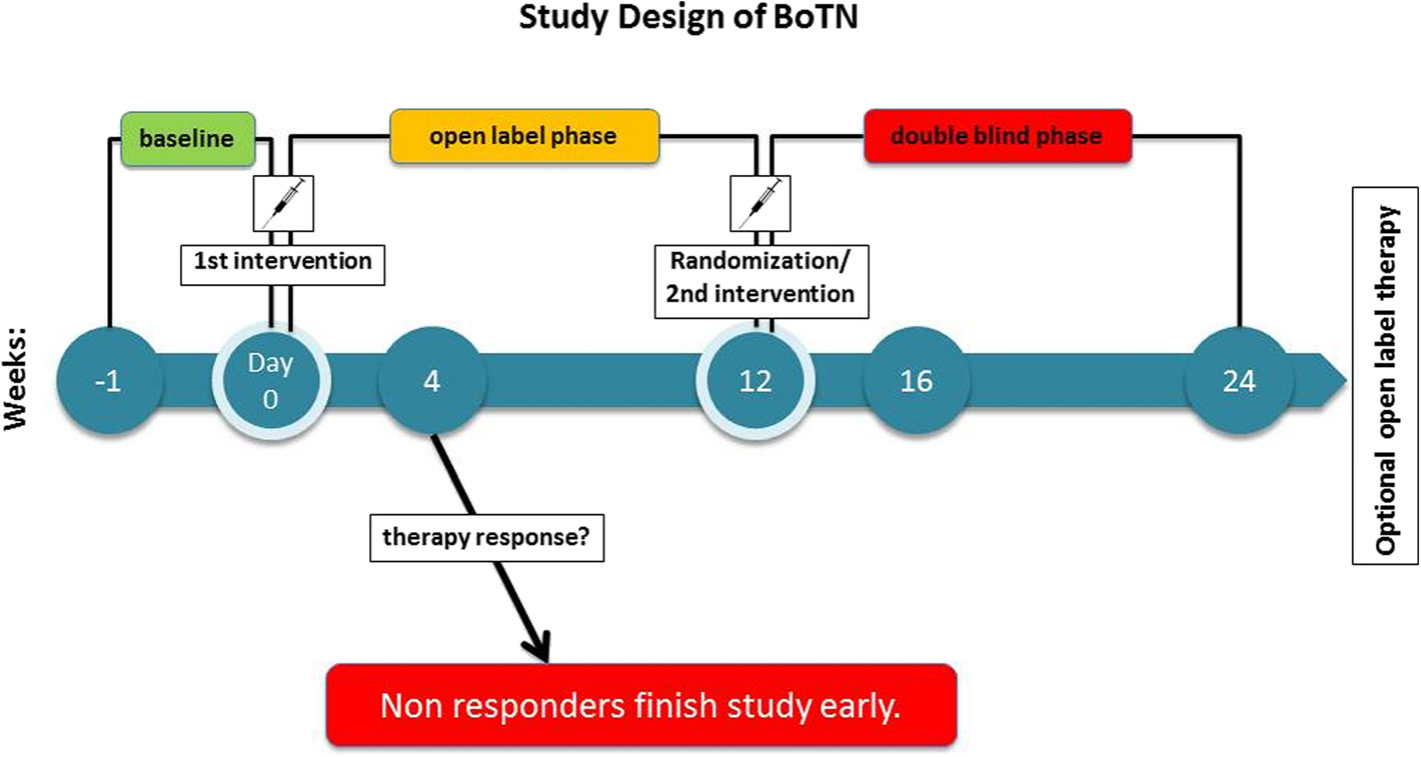
Flow chart of Botulinum neurotoxin type A in the treatment of classical Trigeminal Neuralgia (BoTN)

**Fig. 2:**
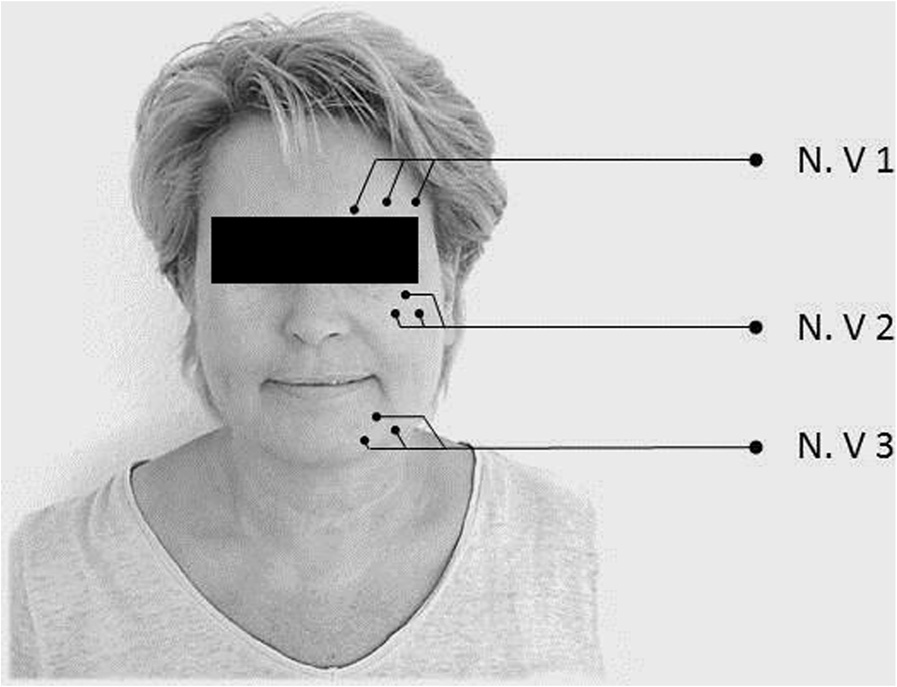
Injection scheme of Botulinum neurotoxin type A in the treatment of classical Trigeminal Neuralgia (BoTN). Nerve exits of the affected trigeminal branches will be palpated and identified. Five units of BT-A are injected at three sites per branch 1.5 cm apart. Injection sites of the first trigeminal branch are kept 1.5 cm above the eyebrows in order to prevent ptosis

Double blind phase (weeks 13–24): therapy responders will be randomized to BT-A injections or placebo injection (saline) at the same injection sites. Allocation to the *verum*/placebo arm is double blind: the BT-A will be dissolved in saline in a 0.5 ml/1 ml syringe and 5 U will be injected near to the nerve exits of the affected trigeminal branches (Figs. 1 and 2).

### Primary outcome parameter

Primary outcome parameter is the average number of TN attacks within the fourth week after double blind intervention in V2.

### Secondary outcome parameters

1. Change in average TN attack frequency in the fourth week after BT-A/placebo injection in V2 (= week 16) as compared to the last 7 days before V0 and V2.
2. Change in average TN attack frequency in the 2nd, 6th, 8th and 12th weeks after V2 as compared to the last 7 days before V0 and V2.
3. Average pain intensity scores (measured on an 11-point NRS) in the 2nd, 4th, 6th, 8th and 12th weeks after V2 as compared to the last 7 days before V0 and V2.
4. Total number of TN attacks in comparison to baseline and V1.
5. Tolerability and safety: adverse events, abnormal neurological and clinical findings assessed in V1–4, Clinical Global Impression (CGI) assessed in V1–4 [39].
6. Rescue analgesic medication needed as compared to placebo and run-in phase.
7. Number of days with TN attacks after V2.
8. Therapy response (reduction of TN attacks per day ≥ 30 %) in 4th, 8th and 12th therapy weeks as compared to baseline and in 16th, 20th and 24th weeks as compared to baseline and 12th therapy week
9. Quality of life/impairment in everyday life assessed in V0, V2 and V4 by a) 12-item short form questionnaire (SF-12), b) Headache Impact Test (HIT-6), c) Allgemeine Depressionsskala: General Depression Scale (ADS) [40–42]

### Concomitant drug therapy

#### Prophylactic therapy

Medical prophylactic therapy of TN can be administered as long as dosages remain consistent from 4 weeks prior to the beginning and throughout the course of the study.

The following drugs are permitted, with the respective maximum dosages and under the surveillance of potential side effects: CBZ, OXC, baclofen, phenytoin, gabapentin, lamotrigine, levetiracetam, pregabalin, topiramate und valproic acid.

Opioids and tricyclic antidepressants (e.g. amitriptyline) can potentially alter TN attack frequency and are, therefore, only permitted when the dosage remains unchanged from 4 weeks prior to the beginning of the study and throughout the course of the study.

#### Rescue pain medication

The following analgesic substances and their maximal daily doses are permitted throughout the course of the study, their use should be documented in the patient’s diary.

- Ibuprofen max. 800 mg/d
- Acetylsalicylic acid max. 1000 mg/d
- Indomethacin max. 50 mg/d
- Diclofenac max. 50 mg/d
- Naproxen max. 250 mg/d
- Paracetamol max. 1000 mg/d
- Metamizol max. 2000 mg/d
- Mixed analgesics, i.e. Thomapyrin (acetylsalicylic acid 250 mg, paracetamol 200 mg and caffeine 50 mg) 2 tablets per day.

### Visit scheme

#### Screening visit (“visit −1”)

In the screening visit a standardized interview is performed: eligibility is assessed and informed consent obtained. Subjective burden will be evaluated with three standardized questionnaires addressing quality of life (SF-12), depression (ADS) and headache severity headache (HIT-6).

Medical and neurological examinations are performed and blood samples are obtained in order to screen for acute infections and disorders of hemostasis. A pain diary is handed out for the assessment of the patient`s baseline TN attack frequency.

#### Visit 0 (baseline)

This visit is scheduled 8 days after the screening visit. Eligible study patients with an average of three or more TN paroxysms per day during baseline phase are treated with BT-A injections in a single blinded manner (for further details see “blinding process” below).

#### Visit 1

Visit 1 is performed between 28 and 31 days after V0 and consists of a standardized, interview as well as medical and neurological examination. ADS, HIT-6 and SF-12 scores are reassessed and adverse events documented. Patients with insufficient therapy response finish the study early.

#### Visit 2

Visit 2 (between 84 and 87 days after V0) consists of the second, this time double blind, placebo controlled, intervention and in addition to that neurological and medical examination and assessment of adverse events and ADS, HIT-6 and SF-12 scores.

#### Visit 3

V3 is comparable to V1 and is scheduled 28–31 days after V2. A standardized interview, medical and neurological examination, assessment of ADS, HIT-6 and SF-12 scores as well as documentation of all adverse events are performed.

#### Visit 4

V4 (between 84 and 87 days after V2) is the final visit and includes neurological and medical examination and assessment of adverse events (including lab work) and ADS, HIT-6 and SF-12 scores. Responders to BT-A therapy are offered continuing BT-A injections as off-label-treatment, if health care providers offer reimbursement of the therapy costs.

### Laboratory testing

Laboratory testing will be performed in V1 including: alanine transaminase, aspartate transaminase, gamma-glutamyl transpeptidase, hemoglobin, white blood cell count, platelet count, creatinine, c-reactive peptide, prothrombin time/international normalized ratio (INR), and partial thromboplastin time.

### Additional neurophysiological testing

**Fig. 3:**
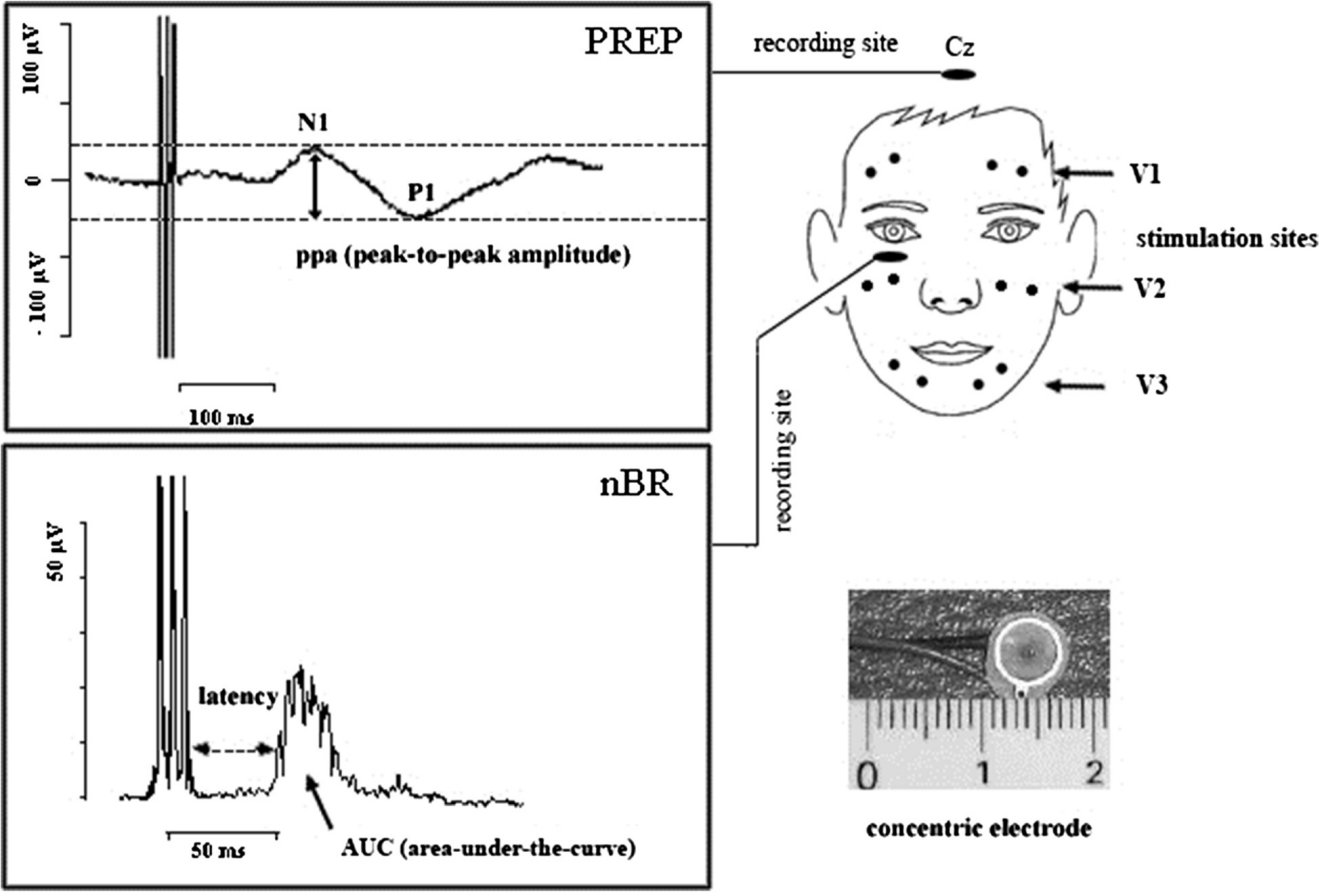
Nociceptive blink reflex and pain- related evoked potentials

The analgesic mechanisms of BT-A on the trigeminal nociceptive system will be further examined by neurophysiological tests: before visits V0–V4 nociceptive blink reflex and pain-related evoked potentials will be recorded using a concentric electrode which generates a series of short painful stimuli over nerve branch V1 of the affected side. This method is established in the testing of trigeminal nociceptive fiber function and the evaluation of supraspinal trigeminal pain processing and central facilitation. It has been used in studies of TN as well as other headache disorders and has no known serious side effects [43–46]. For more information on recording technique, background information and informative value please see reference [47, 48] (Fig. 3).

### Randomization/blinding process

Before participation in the trial patients are informed that in the two scheduled interventions they will receive at least once a *verum* injection with BT-A.

The first intervention in V1 is single blind: BT-A is dissolved in 0.9 % saline and filled in 1- ml injection syringes. This process is not visible to the patient.

The second intervention in V2 is double blind and, therefore, the process of reconstitution will be performed in the pharmacy of the University Hospital Essen:

- Prior to the intervention (<24 hours) a pharmacist, who is not further involved in the conduction and/or analysis of the study dissolves either BT-A in 1 ml of 0.9 % saline or fills the 1-ml injection syringes solely with 0.9 % saline. This process is not visible either to the patient or to any member of the study group
- The allocation to the *verum* or placebo group is done in a pseudorandomized manner: a randomization list assigns screening numbers of patients to either the placebo or *verum* group. This list is only accessible to the pharmacist who is responsible for the reconstitution of BT-A. The screening number is handed out to the pharmacy prior to the intervention by a member of the study group.

### Eligibility

#### Inclusion criteria

- Men and women age 18 years or more, of legal competence and with sufficient knowledge of written and spoken German, capable of presenting themselves to regular visits
- Diagnosis of classic TN as defined in the International Classification of Headache Disorders, third edition (ICHD-3 beta)
- Baseline TN frequency of at least 3 paroxysms per day for < 15 days/month
- At least 1 treatment attempt with CBZ and either insufficient therapy response under a daily dosage of at least 600 mg/d or intolerable side effects
- Stable dosage of concomitant TN drug therapy in the last 4 weeks before participation in the study
- Stable dosage of concomitant drug therapy with potential influence on TN frequency and pain intensity (tricyclic antidepressants, opioids).

#### Exclusion criteria

- Symptomatic painful trigeminopathies/symptomatic TN
- History of trigeminoautonomic headache syndromes:
  - Short-lasting Unilateral Neuralgiform headache with Conjunctival injection and Tearing (SUNCT)
  - Short-lasting Unilateral Neuralgiform headache with conjunctival injection and Autonomic features (SUNA)
  - Paroxysmal hemicrania
  - Cluster headache
- Known sensitivity/intolerance to BT-A
- Contraindications against Botox® according to summary of product characteristics: www.allergan.com
- Local inflammation at injection site
- Impaired hemostasis, anticoagulatory medication
- Known motor neuron or neuropathic disease
- Impairment of neuromuscular transmission
- History of dysphagia
- Corneal ulceration
- Severe allergic diathesis
- Terminal or psychiatric comorbidities
- Pregnancy
- Nursing period
- History of alcoholism/substance abuse

### Excluded therapies and medications

Concomitant medication/non-medical treatment will be documented. The following substances which can potentially alter the efficacy of BT-A cannot be taken during the study and need to be stopped at least 10 days prior to the beginning of the study:

- Spectinomycin
- Aminoglycosides
- Substances blocking neuromuscular transmission, e.g. non-depolarizing muscle relaxants, succinylcholine, dantrolene

Anticoagulatory drugs cannot be taken during the study and need to be stopped at least 10 days prior to the beginning of the study:

- Oral anticoagulants (dabigatran, rivaroxaban, abixaban, coumarin-derivatives)
- Heparin and low molecular weight heparins in effective anticoagulatory dosage

### Assessment of representativeness

All patients screened for BoTN will be registered and documented (date, age, sex, reason for exclusion) in order to assess representativeness of the included patient group.

Patients can always withdraw their consent to participate in the study without stating a reason.

### Statistical analysis and sample size considerations

#### Definition of study populations

### Safety assessment group

Every patient who received BT-A at least once will be included in the safety assessment group. Patients who quit the study early should still participate in the following visits for safety assessment.

### ITT population

The intention-to-treat-analysis (ITT) population will consist of patients who have received study medication once, but analyses will be performed with respect to randomization allocation.

### Randomized ITT population

All responders in the first single blinded part of the trial will be included in the analysis of the randomized ITT population.

### PP population

The per-protocol (PP) population excludes patients with protocol violations such as violation of inclusion/exclusion criteria or illegitimate changes in concomitant medication. Prior to unblinding the sponsor and the responsible biometrician will determine whether the patient will be allocated to PP-analysis or ITT-analysis.

#### Efficacy

The primary endpoint in the efficacy assessment is the average number of TN paroxysms within the fourth week after double blind intervention in V2 (the last 4 days before V3). The confirmatory analysis of the primary endpoint will be the comparison of the *verum* (μ_Botox_) and placebo group (μ_placebo_) with the following, formal hypotheses:

$$ {H}_0:\kern1.5em {\mu}_{Botox}={\mu}_{placebo} $$

$$ {H}_1:\kern1.5em {\mu}_{Botox}\ne {\mu}_{placebo} $$

The confirmatory testing will be performed with the randomized ITT population.

#### Sensitivity analysis

Due to the small sample size further subgroup analysis will be limited to separate assessment of the female and male patient population.

Secondary endpoints will undergo explorative and descriptive analysis – e.g. in the ITT and PP populations. Details will be described in a statistical analysis plan.

#### Safety

All patients of the safety assessment group will take part in the analysis of tolerability and safety. The assessment is part of the screening and all follow-up visits and contains: medical and neurological examination and grading of tolerability on a four-point scale. Adverse events and suspected adverse effects will be separately documented in the Case Report Form (CRF) including duration, severity, their possible relation to the study medication, medical measures taken and its outcome. Severe adverse events will be published as single case reports.

#### Sample size and power

Primary endpoint is the average number of TN paroxysms within the fourth week after double blind intervention. The effect size is estimated at 1.2 and the sample size is calculated by using the Wilcoxon-Mann-Whitney test (software used is G® Power Version 3.1.9.2.).

With *α* = 0.05 and *β*-error = 0.80 the expected sample size per group is *n* = 14, respectively n = 17 considering the expected drop-out rate of 20 %.

Overall a total of 34 recruited patients is expected to be necessary and at least 28 patients need to enter the randomized double blind arm of the trial. Further patients will be recruited if there are more non-responders to treatment as estimated.

#### Quality assurance/monitoring

Due to the small size of this study the sponsor disclaims external monitoring. The sponsor is held responsible for the surveillance of this study and monitoring activity (e.g. source data verification, assessment of protocol violations) will be performed by a person (monitor) with the adequate qualification who is not further involved in the execution and analysis of this trial. All patients will be monitored.

Every member of the study group has received the necessary training in the conduction of this specific trial and is adequately qualified in accordance with Good Clinical Practice (GCP).

### Informed consent, ethics, data safety monitoring board

#### Informed consent

A written informed consent must be obtained from all patients before the trial. This will also be documented in the clinical file. Informed consent includes general characteristics of the study, alternative treatment options, potential risks and benefits from the study, as well as the right to withdraw consent at any given time.

#### Ethics/legal approval

All procedures of this study are in accordance to the German legal regulations of the Medicinal Products Act (AMG), the Declaration of Helsinki and the current version of the International Conference on Harmonization-GCP (ICH-GCP) guidelines. The conduct of this trial, as well as its study protocol have been approved by the responsible ethics committee of the university of Duisburg-Essen, as well as by the responsible surveilling authorities Bundesinstitut für Arzneimittel und Medizinprodukte, the Federal Institute for Drugs and Medical Devices (BfARM).

#### List of the responsible ethics committees

The Ethics Committee of the Medical Faculty of the University of Duisburg-Essen, see also Additional file 1 (“List of the responsible ethics committee and surveilling authorities”) for further details.

## Discussion

BoTN investigates the efficacy and safety of local BT-A injections in the treatment of otherwise therapy-refractory TN in addition to concomitant standard drug treatment. All eligible patients will be first treated with *verum* in a single blinded manner. Responders will be randomized 1:1 to a second intervention that is double blind, placebo controlled (randomized withdrawal design).

Local BT-A injections may prove to be a potentially safe and efficient additional treatment option for severe cases of TN. Additional neurophysiological testing may provide further insight into the antinociceptive mechanisms of BT-A.

### Trial status

Patient recruitment has begun and is still ongoing.

### Consent

Written informed consent was obtained from the patient(s) for publication of this manuscript and accompanying images. A copy of the written consent is available for review by the editor-in-chief of this journal.

## Additional file

Additional file 1:
**List of the responsible ethics committee and surveilling authority.** List includes name and address of the responsible ethics committee and the responsible federal surveilling authority. (DOCX 15 kb)

## Abbreviations

ADS: Allgemeine Depressionsskala: General Depression Scale – a psychometric self-assessment screening test for depression
AMG: German legal regulations of the Medicinal Products Act
BfARM: Bundesinstitut für Arzneimittel und Medizinprodukte, Federal Institute for Drugs and Medical Devices
BMBF: German Ministry of Education and Research
BT-A: botulinum toxin type A
CBZ: carbamazepine
CGI: Clinical Global Impression – a scale that helps evaluating effects of therapeutic intervention, regarding severity of disease, improvement efficacy and adverse effects of therapy
CRF: Case Report Form
GCP: Good Clinical Practice
HIT-6: Headache Impact Test-6 – a 6-item questionnaire for screening and monitoring outcomes of patients with headache
ICH: International Conference on Harmonization
INR: international normalized ratio
ITT: intention-to-treat, NRS, numeric rating scale
OXC: oxcarbazepine
PP: per-protocol
RCT: randomized controlled trial
SF-12: 12-item short form questionnaire: a questionnaire to assess quality of life
SUNA: Short-lasting Unilateral Neuralgiform headache with conjunctival injection and Autonomic features
SUNCT: Short-lasting Unilateral Neuralgiform headache with Conjunctival injection and Tearing
TN: trigeminal neuralgia
VAS: visual analogue scale
ZKSE: Zentrum für Klinische Studien Essen: Center for Clinical Trials Essen
