# Supplementary material for: Botulinum neurotoxin type A in the treatment of classical Trigeminal Neuralgia (BoTN): study protocol for a randomized controlled trial
Source: Trials. 2015 Dec 3;16:550. doi: 10.1186/s13063-015-1052-z (PMC4669653; doi:10.1186/s13063-015-1052-z)
Supplement: Additional file 1: — List of the responsible ethics committee and surveilling authority. List includes name and address of the responsible ethics committee and the responsible federal surveilling authority. (DOCX 15 kb) [file 13063_2015_1052_MOESM1_ESM.docx]

**List of the responsible ethics committee and surveilling authority**

1. Ethik-Kommission der Universität Essen-Duisburg

Robert-Koch-Str. 9-11

45147 Essen

1. **Bundesinstitut für Arzneimittel und Medizinprodukte**
   Kurt-Georg-Kiesinger-Allee 3

53175 Bonn
